# YtrA_Sa_, a GntR-Family Transcription Factor, Represses Two Genetic Loci Encoding Membrane Proteins in *Sulfolobus acidocaldarius*

**DOI:** 10.3389/fmicb.2019.02084

**Published:** 2019-09-10

**Authors:** Liesbeth Lemmens, Laurentijn Tilleman, Ezra De Koning, Karin Valegård, Ann-Christin Lindås, Filip Van Nieuwerburgh, Dominique Maes, Eveline Peeters

**Affiliations:** ^1^Research Group of Microbiology, Department of Bioengineering Sciences, Vrije Universiteit Brussel, Brussels, Belgium; ^2^Laboratory for Pharmaceutical Biotechnology, Faculty of Pharmaceutical Sciences, Ghent University, Ghent, Belgium; ^3^Molecular Biophysics, Department of Cell and Molecular Biology, Uppsala University, Uppsala, Sweden; ^4^Department of Molecular Biosciences, The Wenner-Gren Institute, Stockholm University, Stockholm, Sweden; ^5^Structural Biology Brussels, Department of Bioengineering Sciences, Vrije Universiteit Brussel, Brussels, Belgium

**Keywords:** archaea, *Sulfolobus*, transcription regulation, GntR, YtrA, membrane protein

## Abstract

In bacteria, the GntR family is a widespread family of transcription factors responsible for the regulation of a myriad of biological processes. In contrast, despite their occurrence in archaea only a little information is available on the function of GntR-like transcription factors in this domain of life. The thermoacidophilic crenarchaeon *Sulfolobus acidocaldarius* harbors a GntR-like regulator belonging to the YtrA subfamily, encoded as the first gene in an operon with a second gene encoding a putative membrane protein. Here, we present a detailed characterization of this regulator, named YtrA_Sa_, with a focus on regulon determination and mechanistic analysis with regards to DNA binding. Genome-wide chromatin immunoprecipitation and transcriptome experiments, the latter employing a *ytrA*_*Sa*_ overexpression strain, demonstrate that the regulator acts as a repressor on a very restricted regulon, consisting of only two targets including the operon encoding its own gene and a distinct genetic locus encoding another putative membrane protein. For both targets, a conserved 14-bp semi-palindromic binding motif was delineated that covers the transcriptional start site and that is surrounded by additional half-site motifs. The crystallographic structure of YtrA_Sa_ was determined, revealing a compact dimeric structure in which the DNA-binding motifs are oriented ideally to enable a specific high-affinity interaction with the core binding motif. This study provides new insights into the functioning of a YtrA-like regulator in the archaeal domain of life.

## Introduction

The GntR family of transcription factors (TFs), named after a gluconate operon repressor first characterized in *Bacillus subtilis* (Fujita et al., 1986), is a widespread and abundant TF family amongst prokaryotes (Haydon and Guest, 1991; Rigali et al., 2002; Hoskisson and Rigali, 2009; Suvorova et al., 2015). In bacteria, it is the fourth largest TF family representing about 8% of all regulators (Pérez-Rueda et al., 2018). Members of the GntR superfamily regulate a variety of biological processes, for example metabolic pathways, morphogenesis, sporulation, cell envelope stress response or the production of secondary metabolites such as antibiotics (Rigali et al., 2002; Hillerich and Westpheling, 2006; Hoskisson and Rigali, 2009; Ostash et al., 2011; Salzberg et al., 2011). GntR-type TFs harbor a DNA-binding domain at their N-terminus with a typical helix-turn-helix (HTH) motif that is characterized by a highly conserved secondary structure, despite an overall low sequence identity (Rigali et al., 2002). In addition to the structurally conserved N-terminal domain, a divergent effector-binding/oligomerization domain (E-O domain) can be discerned at the C-terminus. Effector binding typically induces conformational changes in the dimeric protein, thereby affecting binding to a palindromic binding motif (Rigali et al., 2002; Gao et al., 2007). The E-O domain shows a large variability in terms of length, structure and oligomerization mechanism. Based on this heterogeneity in the E-O domain, GntR-type TFs are further classified in at least six subfamilies: FadR, MocR, HutC, YtrA, AraA, and PlmA (Rigali et al., 2002; Suvorova et al., 2015).

YtrA is a GntR subfamily that is prototyped by the YtrA TF of *Bacillus subtilis*, which regulates acetoin utilization and is responsive to cell wall antibiotics suggesting that is involved in cell envelope stress response (Yoshida et al., 2000; Salzberg et al., 2011). Although the YtrA subfamily is the least represented of all GntR subfamilies in bacterial genomes (Vindal et al., 2007), an evolutionary expansion has been observed in certain bacterial phyla, such as Gram-positive Firmicutes and Actinobacteria (Suvorova et al., 2015), with the latter having multiple YtrA homologs in a single genome (Tsypik et al., 2016). The actinomycete *Streptomyces coelicolor* harbors several YtrA-like TFs with likely at least partially overlapping functions, for example in controlling genes affecting morphogenesis and antibiotic production (Hillerich and Westpheling, 2006; Tsypik et al., 2016). YtrA-like TFs are generally encoded as the first gene in an operon also encoding an ATP-binding cassette (ABC) transport system (Rigali et al., 2002; Hoskisson and Rigali, 2009). By repressing transcription of this operon, YtrA regulators simultaneously target their own transcription and that of the co-encoded ABC transport system. A phylogenomic study in actinomycetes revealed that in this bacterial clade, in which YtrA-like TFs are abundant, exceptions exist with respect to the genomic colocalization of YtrA-encoding genes and genes coding for ABC transport systems (Tsypik et al., 2016). More specifically, one group of YtrA-like TFs was characterized by an operonic colocalization with genes predicted to encode membrane proteins (Tsypik et al., 2016). The function of these membrane proteins and the role of their regulation by a YtrA regulator remains unclear (Tsypik et al., 2016).

YtrA members, with a total average length of 120 to 130 amino acids, are typified by a relative small E-O domain, with approximately 50 amino acids folding into two α-helices (Rigali et al., 2002). Structural analysis of a YtrA-like TF of *Corynebacterium glutamicum* demonstrated that these α-helices mediate dimerization of the protein (Gao et al., 2007). Despite the small size of the E-O domain, the dimerization mode resulted in an extended global structure, with the two monomeric HTH motifs being separated by a relatively large distance (Gao et al., 2007). Such a structure is in line with the observation that the palindromic binding motif is longer for members of the YtrA subfamily with respect to other subfamilies (Rigali et al., 2002; Vindal et al., 2007). Indeed, the YtrA binding motif is typically 24 base pairs (bp) long and characterized by distantly located half sites harboring conserved palindromic residues that are contacted by the respective HTH motifs (Rigali et al., 2002; Gao et al., 2007). Given the small E-O domain it was hypothesized that YtrA-like regulators might not be capable of forming a ligand-binding pocket (Yoshida et al., 2000; Rigali et al., 2002). Although this hypothesis was refuted by the observation of 2-methyl-2,4-pentanediol (MPD) molecules interacting in the E-O domain of the crystallized *C. glutamicum* YtrA-like protein (Gao et al., 2007), MPD does not allosterically affect this regulator and, moreover, a biologically relevant ligand has not yet been identified for a YtrA member (Gao et al., 2007; Salzberg et al., 2011).

Archaea harbor, despite being characterized by a eukaryotic-like transcription machinery (Bell and Jackson, 2001), TFs belonging to typical bacterial TF families (Pérez-Rueda and Janga, 2010). Phylogenomic predictions indicate that TFs of the GntR family can also be recognized in archaeal genomes, although they represent only 1.3% of the total predicted TF repertoire and are thus significantly less abundant as compared to bacteria (Pérez-Rueda et al., 2018). Despite representing only a minor proportion of archaeal TFs, several GntR-like regulators are predicted in both Eury- and Crenarchaeota (Pérez-Rueda et al., 2018). To our knowledge, no archaeal GntR-like transcriptional regulator has been experimentally described. It is unclear whether the physiological functions and molecular mechanisms in archaea are reminiscent of the bacterial GntR members. The thermoacidophilic model crenarchaeote *Sulfolobus acidocaldarius* is predicted to harbor a gene encoding a putative GntR-type TF belonging to the YtrA subfamily. Here, we aim at characterizing this TF, named YtrA_Sa_, by unraveling its structure, DNA-binding characteristics and regulon.

## Materials and Methods

### Bioinformatic Analysis

Homology searches were performed using Standard Protein BLAST (National Center for Biotechnology Information) and SyntTax (Oberto, 2013). Multiple sequence alignments were constructed with MUSCLE (Edgar, 2004). Archaeal genomes were explored using the UCSC Archaeal Genome Browser (Chan et al., 2012). The RSAT suite was used for the prediction of binding motifs (Nguyen et al., 2018). Sequence logos were prepared with WebLogo (Crooks et al., 2004).

### Microbial Strains and Growth Conditions

*Sulfolobus acidocaldarius* strains were cultivated in basic Brock medium (Brock et al., 1972) supplemented with (w/v) 0.2% sucrose and (w/v) 0.1% N-Z-amine. The pH of the medium was adjusted to 3.5 using sulfuric acid. Microbial growth was performed at 75°C in a shaking incubator and was followed by measurement of optical density at 600 nm (OD_600_). For cultivation of the uracil auxotrophic strains MW001 (Wagner et al., 2012) and SK-1 (Suzuki and Kurosawa, 2016), the growth medium was supplemented with 10 mg/ml uracil. To induce expression under the control of P*mal*, 0.4% maltose was added to the growth medium. For growth on solid medium, Brock medium was solidified by the addition of 0.6 (w/v)% gelrite, 10 mM MgCl_2_ and 3 mM CaCl_2_.

*Escherichia coli* strains were used for propagation of plasmid DNA constructs and heterologous protein overexpression and were grown while shaking at 37°C in Lysogeny Broth (LB) medium supplemented with 50 μg/ml ampicillin, 50 μg/ml kanamycin, and/or 34 μg/ml chloramphenicol, depending on the used strain and plasmid construct. An overview of all strains used in this work is given in Supplementary Table S1.

### Nucleic Acid Manipulations and Molecular Cloning

For genomic DNA (gDNA) extractions, *S. acidocaldarius* cells were cultivated to an OD_600_ of 0.8, followed by centrifugation at 7000 rpm and a magnetic-bead based purification using a QuickPick^TM^ SML gDNA kit (Bio-Nobile) according to manufacturer’s instructions. For RNA extractions, cultures were grown until reaching an OD_600_ of 0.4, after which 4-ml samples were treated with RNAprotect Bacteria Reagent (Qiagen) and pelleted. Total RNA was purified using an RNeasy Mini Kit (Qiagen) with an additional on-column DNase I treatment using the RNase-free DNase set (Qiagen). RNA samples were tested for integrity using RNA gel electrophoresis and stored at −80°C until further analysis.

Using *S. acidocaldarius* gDNA, the YtrA_Sa_-encoding gene, *Saci_1851*, was amplified with oligonucleotides LL106 and LL107 and cloned into a pET24a expression vector by traditional restriction-ligation cloning using the NdeI and XhoI restriction sites. An overview of all oligonucleotide sequences and plasmid constructs used in this work is presented in Supplementary Tables S2, S3, respectively. Cloning was performed in *E. coli* using heat-shock transformations.

A *Sulfolobus*/*E. coli* shuttle vector plasmid enabling to overexpress *ytrA*_*Sa*_ in *S. acidocaldarius* was generated in multiple steps in *E. coli*, starting from the pRN1-derived pSVA1450 (Wagner et al., 2012). In a first step, vector pJL1602 was constructed by replacing the maltose-inducible promoter P*mal* upstream of *lacS* by a synthetic expression region harboring a multiple cloning site (MCS) (consisting of 5 unique restriction sites NdeI, NotI, SalI, PstI, and NheI) flanked by two constitutive promoters (natural promoters of Sso0389 and Sso1927, respectively). To this end, a ligase chain reaction approach was performed for oligonucleotides EP479-EP494 (Supplementary Table S2) followed by cloning using the Seamless Ligation Cloning Extract (SLiCE) technique (Zhang et al., 2012). Linearization of the plasmid was performed by SacII and NcoI restriction, while the insert fragment was prepared by amplifying the synthetic expression region using primers EP495 and EP496 (Supplementary Table S2). Next, the Sso0389 promoter was replaced by P*mal* with a traditional cloning approach using SacII and SalI restriction sites, thereby generating pLL1601. Finally, the *Saci_1851* gene was PCR-amplified from *S. acidocaldarius* gDNA and cloned into the MCS downstream of P*mal* using SdaI and BspOI restriction sites, thus generating pLL1601***x****ytrA*_*Sa*_.

The YtrA_Sa_-overexpression construct pLL1601***x****ytrA*_*Sa*_ was transformed into *S. acidocaldarius* SK-1 as described (Suzuki and Kurosawa, 2016), using a Gene Pulser II electroporator (Bio-Rad) with parameter setting 1.5 kV, 25 μF and 600 Ω and with 1-mm cuvettes (Bio-Rad). Overexpression of *ytrA*_*Sa*_ was confirmed in the presence of maltose using a quantitative reverse transcriptase PCR (RT-qPCR) approach as described below with primers LL021 and LL022 (Supplementary Table 2 and Supplementary Figure S1). Growth was not affected by *ytrA*_*Sa*_ overexpression.

### Heterologous Ytr_Sa_ Expression and Protein Purification

After transforming *E. coli* Rosetta (DE3) with pET24a***x****ytrA*_*Sa*_, the strain was cultivated in liquid medium until reaching an OD_600_ of 0.6, followed by induction with 1 mM isopropyl-β-D-1-thiogalactopyranoside (IPTG) and further cultivation during about 16 h. Subsequently, cells were harvested by centrifugation, resuspended in lysis buffer (50 mM Na_2_HPO_4_, 0.3 M NaCl, pH 8.0) and lysed by sonication. Lysed cells were centrifuged and the soluble phase containing heterologously expressed YtrA_Sa_ protein was collected. Next, this C-terminally His-tagged recombinant protein was purified by affinity chromatography using an ÄKTA-fast protein liquid chromatography (FPLC) system with a 5-ml His-Trap FF column (GE Healthcare). Fractional elution was accomplished by setting a linear gradient between buffer A (20 mM Na_2_HPO_4_, 0.5 NaCl, 40 mM imidazole, pH 7.4) and buffer B (20 mM Na_2_HPO_4_, 0.5 NaCl, 500 mM imidazole, pH 7.4). YtrA_Sa_-containing fractions were analyzed by sodium dodecyl sulfate-polyacrylamide gel electrophoresis (SDS-PAGE), followed by dialysis into a storage buffer (50 mM Na_2_HPO_4_, 150 mM NaCl, pH 7.4).

For the preparation of selenomethionine (SeMet)-substituted YtrA_Sa_ protein, a metabolic inhibition protocol was performed (Lejon et al., 2008). To this end, 2.5-ml culture of *E. coli* B834 (DE3/pLysS) cells transformed with pET24a***x****ytrA*_*Sa*_ was grown during 3 h in LB medium supplemented with kanamycin and used to inoculate 45 ml M9 minimal medium (50 mM Na_2_HPO_4_, 3 g/l KH_2_PO_4_, 0.5 g/l NaCl, 1 g/l NH_4_Cl) and 5 ml LB medium, also supplemented with kanamycin. The growth was continued until an OD_600_ of 0.7 was reached. After that, 50 ml of the culture was added to 900 ml M9 medium and 100 ml LB medium supplemented with 2 mM MgSO_4_, 0.1 mM CaCl_2_, 0.4% glucose and 25 mg/ml kanamycin, which was further cultivated until an OD_600_ between 0.4 and 0.6 was reached. Next, following L-amino acids were added: Lys, Phe and Tyr (100 mg/l), Leu, Ile and Thr (50 mg/l) and L-SeMet (Acros Organics; final concentration of 60 ml/l). After 10 min, protein expression was induced by adding 0.4 mM IPTG followed by overnight incubation. The SeMet-substituted protein was purified by employing the same procedure as described above. All YtrA_Sa_ protein preparations were pure, as judged by SDS-PAGE (Supplementary Figure S2A).

### Chromatin Immunoprecipitation

Chromatin immunoprecipitation (ChIP) was performed as previously described (Liu et al., 2016). Triplicates of 20-ml *S. acidocaldarius* SK-1 cultures were incubated until reaching an OD_600_ of about 0.4. Crosslinking was achieved by the addition of formaldehyde at a final concentration of 1%, immediately after the culture was taken out the incubator, followed by an incubation at room temperature while shaking during 5 min. Next, the crosslinking reaction was quenched by adding glycine at a final concentration of 125 mM, again followed by a 5-min shaking incubation at room temperature. The cultures were centrifuged at 7000 rpm for 10 min, washed twice in Phosphate-Buffered Saline (PBS) buffer and dissolved in 1.5 ml IP buffer [50 mM Hepes-KOH (pH 7.5), 150 mM NaCl, 1 mM EDTA, 1% Triton X-100, 0.1% sodium deoxycholate, 0.1% SDS, protease inhibitor cocktail (Roche Applied Science)]. Subsequently, cells were subjected to sonication as described (Nguyen-Duc et al., 2013a) resulting in obtaining DNA fragments with a length ranging between 100 and 800 bp. The ChIP assay itself was done with affinity-purified polyclonal anti-YtrA_Sa_ rabbit antibodies raised against the purified protein (Innovagen). A procedure was performed using Dynabeads M-280 sheep anti-rabbit IgG beads (Life Technologies) (Smollett et al., 2012). As a mock control, a similar sample was prepared without the addition of the Ytr_Sa_-specific antibody preparation. Finally, ChIP-DNA was purified using the IPure DNA extraction kit (Diagenode). The concentration of these ChIP-DNA samples, measured using the Qubit dsDNA High Sensitivity Assay Kit (Thermo Fisher Scientific), was between 0.5 and 3.12 ng/μL.

For high-throughput sequencing analysis, 20 μl of DNA was prepared using the NEBNext Ultra II DNA Library Prep Kit for Illumina (New England Biolabs) according to the manufacturer’s protocol. During this library preparation, 14 PCR cycles were used for all samples. Libraries were quantified by quantitative PCR (qPCR), according to Illumina’s protocol ‘Sequencing Library qPCR Quantification protocol guide’, version February 2011. A DNA 1000 chip (Agilent Technologies) was used to control the library’s size distribution and quality. Libraries were equimolarly pooled and sequencing was performed on a high-throughput Illumina NextSeq 500 flow cell generating 75 bp single reads. Per sample, on average, 30.5 ± 5.0 million reads were generated. First, these reads were trimmed using cutadapt version 1.17 (Martin, 2011) to remove the Illumina adaptor sequences. The trimmed reads were mapped against the *S. acidocaldarius* DSM639 GCA_000012285.1 reference genome sequence (Chen et al., 2005) using Bowtie2 version 2.2.5 (Langmead and Salzberg, 2012). For each sample, a Tag Directory was made with HOMER version 4.10 (Heinz et al., 2010). Mock samples were used as a control for enriched samples in the findPeaks algorithm of the HOMER suite (Heinz et al., 2010). ChIP-sequencing (ChIP-seq) results were visualized using Integrative Genomics Viewer version 2.4.19 (Robinson et al., 2017).

Target-specific validation of ChIP enrichment was performed with qPCR for biological triplicates. To this end, purified ChIP-DNA and mock samples were amplified using a My-iQ^TM^ Single color Real-time PCR system (Bio-Rad) and GoTaq^®^ qPCR Master Mix (Promega) as previously described (Nguyen-Duc et al., 2013b). Primer3Plus software (Rozen and Skaletsky, 2000) was used to design oligonucleotides (Supplementary Table S2) that amplify fragments around the ChIP-seq peak summit regions and with a length between 150 and 200 bp. The efficiency of oligonucleotide pairs was tested using gDNA as a template. Relative enrichment ratios were calculated following the method described in Pfaffl (2001) using an irrelevant genomic region (in the open reading frame (ORF) of *Saci_1336*) as a non-binding reference.

### Electrophoretic Mobility Shift and Footprinting Assays

^32^P-labeled DNA fragments were obtained by PCR using GoTag^®^ Green Master Mix (Promega) and for each fragment, a 5′-end-labeled oligonucleotide and a non-labeled oligonucleotide (Supplementary Table S2). As a template, *S. acidocaldarius* MW001 gDNA was used. Prior to this, labeled oligonucleotides were prepared using [γ-^32^P]-ATP (Perkin Elmer) and T_4_ polynucleotide kinase (Thermo Scientific). In case of 50-bp fragments, hybridization was performed of a ^32^P-labeled oligonucleotide with a non-labeled complementary one. All labeled DNA fragments were purified from a native polyacrylamide gel (6%) after electrophoresis.

Electrophoretic mobility shift assays (EMSAs) were performed as described before (Enoru-Eta et al., 2000) with approximately 0.1 nM ^32^P-labeled DNA fragment, an excess of non-specific competitor DNA and varying protein concentrations. In case of ligand-binding assays, varying concentrations of aromatic compounds (0 mM, 10 mM and 100 mM) were added as well. Binding reactions were prepared in Lrp binding buffer (20 mM Tris-HCl (pH 8.0), 1 mM MgCl_2_, 0.1 mM dithiothreitol (DTT), 12.5% glycerol, 50 mM NaCl, 0.4 mM EDTA) and were incubated at 37°C to equilibrate prior to electrophoresis on native polyacrylamide gels (6%). Densitometry analysis was performed by measuring the integrated density of the free DNA bands using Image J (Abramoff et al., 2004). The apparent equilibrium dissociation constant K*_Dapp_* and Hill coefficient n were calculated by fitting the density data using the Hill equation as described before (Peeters et al., 2007) using GraphPad Prism software.

DNase I footprinting experiments were performed as previously described (Enoru-Eta et al., 2000; Peeters et al., 2004) with Maxam-Gilbert treated samples as sequencing ladders (Maxam and Gilbert, 1980).

### YtrA_Sa_ Crystallization and Structure Determination

Crystallization of SeMet-derivated YtrA_*Sa*_ was performed at 20°C using the hanging-drop vapor diffusion method by mixing equal volumes of protein solution (11 mg/ml) and reservoir solution consisting of 20% (w/v) PEG3350, 0.2 M sodium nitrate and 0.1 M Bis-Tris propane, pH 8.5. Needle-sized crystals were obtained after a few days (Supplementary Figure S2B). The crystals belong to space group P6_2_, with unit-cell parameters a = b = 132.4 Å, c = 39.2 Å and α = β = 90°, γ = 120° and two molecules per asymmetric unit, with a Matthews coefficient of 2.5 Å^3^/Da and 50.5% solvent content. High-resolution data were collected at beamlines ID23-1 and ID29 (European Synchrotron Radiation Facility (ESRF), Grenoble, France) at −173°C and were processed using the XDS package (Kabsch, 2010). The structure of YtrA_Sa_ was determined with the ARP/Warp module of the CCP4i suite (Lamzin et al., 2012). Structure refinement was performed with PHENIX (Echols et al., 2012) and REFMAC (Murshudov et al., 2011). The C-terminal end of chain B (Asn101- Lys121) could not be built due to poor electron density. This C-terminal end also has high B-factors in chain-A. The coordinates were deposited to the Protein Data Bank.

### Gene Expression Analysis

Triplicate cultures of *S. acidocaldarius* SK-1***x***pLL1601***x****ytrA*_*Sa*_ and the isogenic wild type *S. acidocaldarius* SK-1***x***pLL1601 were grown in the presence of 0.4% maltose and subjected to RNA extraction. To initiate library preparation for an RNA sequencing (RNA-Seq) experiment, the concentration and quality of the extracted total RNA were checked using the “Quant-it Ribogreen RNA assay” (Life Technologies) and the RNA 6000 Nano Chip (Agilent Technologies), respectively. Subsequently, 2 μg of RNA was used to perform a ribo depletion using the Ribo-Zero rRNA Removal Kit (Bacteria) (Illumina) according to the manufacturer’s protocol. Depleted samples were used to perform an Illumina sequencing library preparation using the TruSeq Stranded Total RNA Sample Preparation Kit (Illumina). During the library preparation, 9 PCR cycles were used. Libraries were quantified by qPCR, according to Illumina’s protocol “Sequencing Library qPCR Quantification protocol guide,” version February 2011. A High Sensitivity DNA chip (Agilent Technologies) was used to control the library’s size distribution and quality. Libraries were equimolarly pooled and sequencing was performed on a high throughput Illumina NextSeq 500 flow cell generating 75 bp single reads. Per sample, on average, 58 ± 31 million reads were generated. First, these reads were trimmed using cutadapt version 1.17 (Martin, 2011) to remove the Illumina adaptor sequences. Next, the trimmed reads were mapped against the *S. acidocaldarius* DSM639 GCA_000012285.1 reference genome sequence (Chen et al., 2005) using STAR version 2.5.3a (Dobin et al., 2013). RSEM software version 1.2.31 (Li and Dewey, 2011), was used to generate the count tables. Differential gene expression analysis between the wild type and *ytrA*_*Sa*_ overexpression strain was performed using edgeR (Robinson et al., 2010). First, read counts were normalized using edgeR’s standard normalization method. Only genes with a counts per million (cpm) value above 1 in at least three samples were retained. A general linear model was built, and statistical testing was done using the empirical Bayes quasi-likelihood *F*-test. Genes characterized by a false discovery rate (FDR) < 0.01 and a fold change (FC) > 2 were considered as significantly differentially expressed.

For RT-qPCR analysis, first-strand cDNA was prepared from 1 μg of each purified RNA sample by GoScript^TM^ Reverse Transcriptase (Promega). Oligonucleotides were designed using Primer3Plus software (Rozen and Skaletsky, 2000) (Supplementary Table S2) and tested for efficiency using gDNA as a template. RT-qPCR was performed for biological triplicates using a Bio-Rad iCycler and GoTaq^®^ qPCR Master Mix (Promega) as previously described (Nguyen-Duc et al., 2013b). Relative expression ratios were calculated with respect to the expression levels of the reference gene *Saci_1336* (encoding TATA-binding protein) (Pfaffl, 2001).

## Results

### Identification of a YtrA Homolog in *S. acidocaldarius*

**FIGURE 1:**
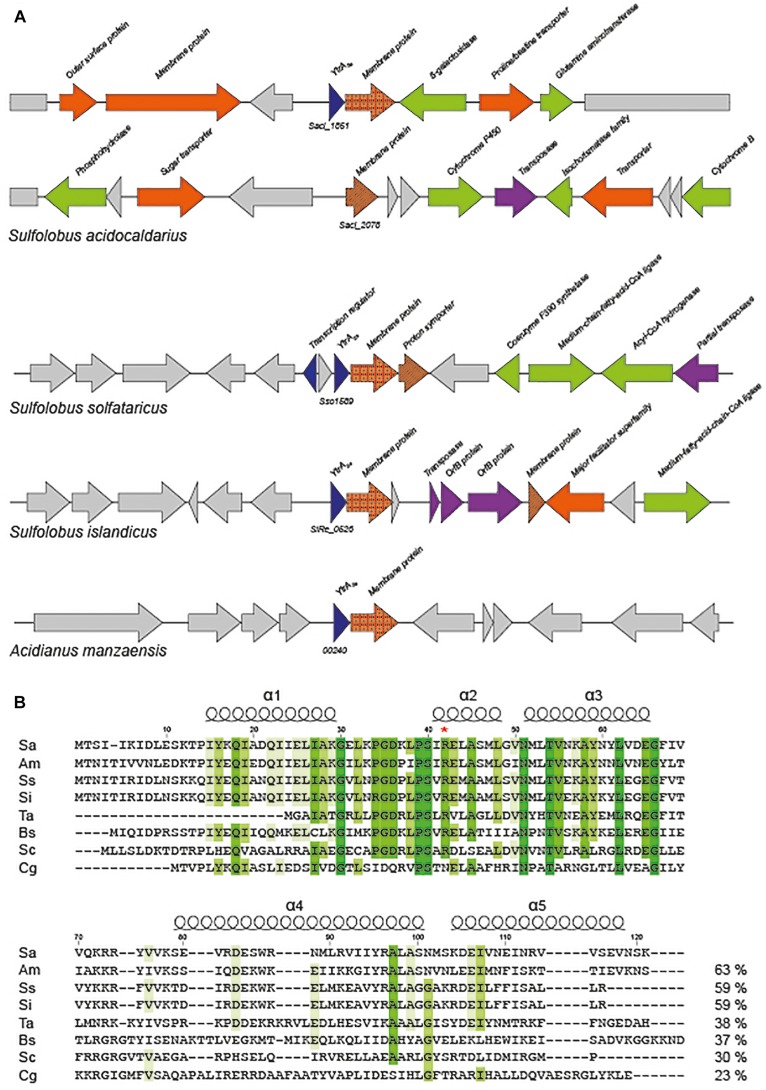
*Sulfolobales* genomes encode a YtrA-like homolog. **(A)** Genomic organization of *ytrA*-like genes in *Sulfolobales*. Gene functions are mentioned based on annotations (Chan et al., 2012) with the following color code: blue, (putative) transcription regulators; orange, (putative) membrane proteins and/or transporters; green, (putative) metabolic enzymes; purple, (putative) transposon-associated functions. All *Sulfolobales* strains of which genome sequences are present in the SyntTax database (Oberto, 2013) harbor a *ytrA* homolog. The strains represented in the scheme are *Sulfolobus acidocaldarius* DSM639, *Sulfolobus solfataricus* P2, *Sulfolobus islandicus* REY15A and *Acidianus manzaensis* YN25. **(B)** Sequence alignment of YtrA-like GntR-family regulators in archaea and bacteria. Following sequences are shown in the alignment: Q4J7S4 *Sulfolobus acidocaldarius* (Sa), A0A1W6JWH0 *Acidianus manzaensis* (Am), Q97XX0 *Sulfolobus solfataricus* (Ss), F0NDQ3 *Sulfolobus islandicus* (Si), Q9HK68 *Thermoplasma acidophilum* (Ta), O34712 *Bacillus subtilis* (Bs), Q9XA65 *Streptomyces coelicolor* (Sc), Q8NLJ5 *Corynebacterium glutamicum* (Cg). Conserved amino acid residues are indicated in shades of green. Sequence identities are provided next to each sequence. Position numbers are indicated with respect to YtrA_Sa_, while secondary structure elements are displayed based on the crystal structure of the *C. glutamicum* YtrA (PDB:2EK5) (Gao et al., 2007). A red asterisk denotes an Arg that is presumed to play an important role in DNA binding.

Based on annotations, it is predicted that the *S. acidocaldarius* genome encodes a putative transcription factor belonging to the GntR family. This protein is only conserved in the closely related *Sulfolobales* species *Acidianus manzaensis*, *Sulfolobus solfataricus*, and *Sulfolobus islandicus* (Figure 1A). Closer inspection of the amino acid sequence encoded by the concerned gene (*Saci_1851*) learned that the C-terminal E-O domain of this 121-amino acid protein consists of about 50 amino acids predicted to fold into two α-helices (Figure 1B), thus classifying it as a member of the YtrA subfamily (Rigali et al., 2002). We, therefore, named this putative transcription factor YtrA_*Sa*_. The *ytrA*_*Sa*_ gene is encoded as the first gene of an operon, a typical feature for YtrA-like TFs. Furthermore, this operon harbors as a second gene a membrane protein-encoding gene (*Saci_1850*), which is conserved as well in the four *Sulfolobales* species (Figure 1A). In addition, the *ytrA* operon in *S. solfataricus* contains a second gene encoding a putative membrane protein, of which a homolog (27% sequence identity on protein level) is encoded on a distant genomic location in *S. acidocaldarius* (*Saci_2078*, Figure 1A).

Sequence comparison with representatives of the different YtrA subfamilies in actinomycetes revealed a clear similarity between YtrA_Sa_ and the subfamily of YtrA-like TFs in *S. coelicolor* that are encoded in an operon with a gene encoding a membrane protein instead of genes coding for an ABC transport system (Figure 1B). The length of the putative α-helix α4 in the C-terminal domain is a major difference between the *Sulfolobales* orthologs and *S. coelicolor* SCO3812 on one hand and other YtrA-like proteins that are co-encoded with an ABC transporter operon. This is predicted to be 4–7 amino acids shorter for the former as compared to YtrA homologs from the Euryarchaeote *Thermoplasma acidophilum* and from the bacteria *B. subtilis* and *C. glutamicum*. Furthermore, for all YtrA proteins, sequence conservation is much lower in the C-terminal E-O domain than in the N-terminal DNA-binding domain (Figure 1B).

### Genome-Wide Interactions Between YtrA_Sa_ and DNA *in vivo*

**FIGURE 2:**
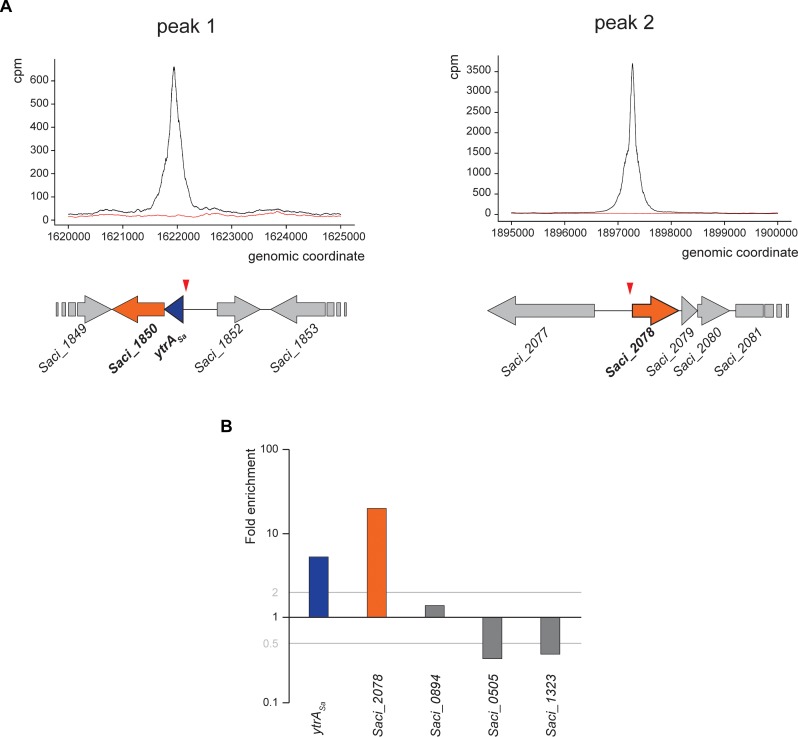
YtrA_Sa_ interacts with two genomic loci *in vivo*. **(A)** Zoomed images of two sections of the genomic binding profile of YtrA_Sa_ as monitored by ChIP-seq, for which enrichment was observed. Below the profile, a schematic representation of the genomic organization of the *in vivo* binding regions is shown with the indication of the ChIP-seq peak summit locations (red triangle). The black curve represents the ChIP sample, while the red curve represents the mock experiment; cpm = counts per million. **(B)** Validation of ChIP enrichment using a qPCR approach with probes designed based on the detected binding region in ChIP-seq (targets are named after the gene located closest in the neighborhood). This experiment has been performed for biological triplicates, with the data shown being representative for all replicate experiments. Fold enrichment is expressed relative to a genomic region within the ORF of *Saci_1336* shown not to be bound by YtrA_Sa_ in the genome-wide ChIP-seq experiment.

As an effort to reveal genomic binding sites of YtrA_Sa_, a ChIP-seq experiment was performed using polyclonal anti-YtrA_Sa_ antibodies (Figure 2 and Table 1). The resulting binding profile consisted of only two highly enriched regions with an average fold enrichment of 24.3 and 113.5 for peak 1 and 2, respectively. The first enriched peak (peak 1) corresponds to a 750-bp region covering the promoter region of the operon encoding the TF itself and a putative membrane protein (*Saci_1851* and *Saci_1850*, respectively) with its summit located close to the transcriptional start of the operon (Figure 2A). A second enriched region of about 800 bp was observed in the intergenic region upstream of another putative membrane protein-encoding gene (*Saci_2078*). Interestingly, this gene was predicted to be homologous to the second membrane protein-encoding gene in the *S. solfataricus* and *S. islandicus ytrA* operons (see above). Similar to peak 1, its summit is located close to the transcriptional start site. Observed enrichments were validated by targeted ChIP-qPCR (Figure 2B). Both qPCR and high-throughput sequencing displayed a higher enrichment for the *Saci_2078* target (peak 2) than for the *Saci_1851* (*ytrA*_*Sa*_) target (peak 1) (targets are named after the gene located closest to the bound genomic region). Besides the two main targets, three additional genomic regions (*Saci_0884*, *Saci_0505* and *Saci_1323* targets) were enriched by ChIP-seq in some, but not all biological replicates. This was not confirmed by ChIP-qPCR (Figure 2B). In conclusion, these data suggest that the YtrA_Sa_ direct regulon is very restricted and limited to only two target regions upstream of genes encoding membrane proteins.

**TABLE 1:** Summary of ChIP-seq results.

**Peak interval (genomic coordinates)**	**Fold enrichment**			**Nearest open reading frame**	**Annotation**	**Peak summit location**
	**R1**	**R2**	**R3**			
1897151–1897397	133.2	96.30	108.0	*ytrA*_*Sa*_	GntR family transcriptional regulator	I
1621827–1622073	30.89	23.72	17.01	*Saci_2078*	Membrane protein	I
718252–718538	7.05	4.64	N.S.	*Saci_0894*	Hypothetical protein	G
419146–419392	4.73	N.S.	N.S.	*Saci_0505*	Conjugative plasmid protein	I
1130433–1130685	N.S.	N.S.	6.47	*Saci_1323*	Hypothetical protein	I

*Fold-enrichments are expressed for three replicate experiments (R1, R2, and R3). It is indicated whether the peak summit is located on an intergenic (I) or intragenic (G) location.*

### *In vitro* Analysis of YtrA_Sa_-DNA Interactions

**FIGURE 3:**
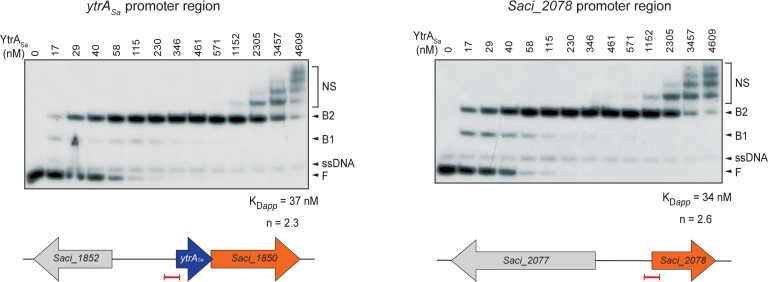
YtrA_Sa_ forms complexes with its DNA targets *in vitro* and with high affinity. Electrophoretic mobility shift assays (EMSAs) of YtrA_Sa_ binding to radiolabeled DNA probes of about 100 bp representing the ChIP-seq peaks. Molar protein concentrations are indicated above the autoradiograph. Populations of free DNA (F), single-stranded DNA (ssDNA), YtrA_Sa_-bound DNA (B1 and B2) and non-specific DNA-protein complexes (NS) are indicated with an arrowhead or accolade. Below, schematic representations are shown of the location of the probe (indicated in red) used in EMSA with respect to the open reading frames (ORFs) in the genomic neighborhood of the target regions. Calculations of apparent dissociation constants (K*_Dapp_*) and Hill coefficients (n) are based on densitometric analysis of free DNA bands followed by binding curve analysis.

For both ChIP-seq enriched regions, electrophoretic mobility shift assays (EMSAs) were performed using purified YtrA_Sa_ protein and labeled DNA probes of about 100 bp each encompassing the center of a ChIP-seq peak (Figure 3). These assays demonstrated high-affinity and specific interactions of YtrA_Sa_ with the DNA *in vitro*, resulting in the formation of two electrophoretically distinct complexes (B1 and B2) at relatively low protein concentrations (between 17 and 230 nM). The observation of a transient formation of complex B1 at low relative abundances suggests that the interaction is characterized by a positive binding cooperativity, which is corroborated by densitometric analysis of EMSA autoradiographs resulting in Hill coefficients of 2.3 and 2.6 for the *ytrA*_*Sa*_ and *Saci_2078* targets, respectively. Furthermore, apparent dissociation constants K*_Dapp_* are estimated to be 37 and 34 nM, respectively (Figure 3). Quantitative and qualitative binding characteristics are thus very similar for both target regions, in contrast to the *in vivo* binding characteristics as observed by the ChIP enrichments.

**FIGURE 4:**
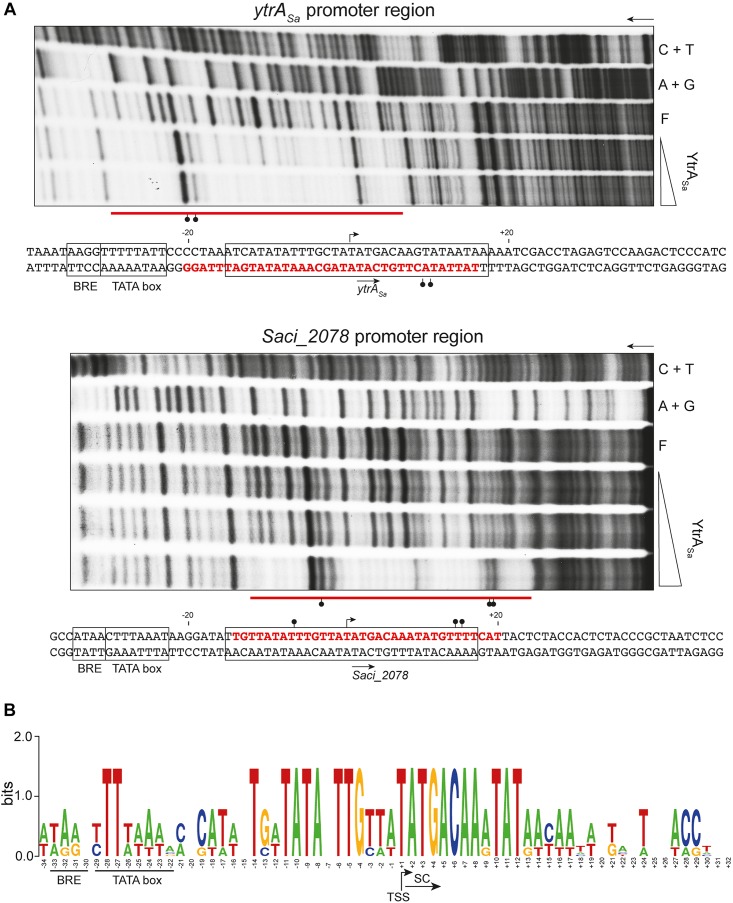
YtrA_Sa_ binds a conserved binding motif covering the transcriptional start for both targets. **(A)** Autoradiographs of DNase I footprinting experiments performed with 102- or 100-bp DNA probes having either the bottom (*ytrA*_*Sa*_ target) or top (*Saci_2078* target) strand ^32^P-labeled, respectively. Arrows indicate the direction of electrophoresis; “A + G” and “C + T” represent the purine- and pyrimidine-specific Maxam-Gilbert sequencing ladders, respectively; “F” denotes the population of free DNA; the other lanes show samples to which YtrA_Sa_ protein was added at the following concentration range: 40–571 nM for the *ytrA*_*Sa*_ target and 40–230 nM for the *Saci_2078* target. Protected zones are indicated with a horizontal red line, while DNase I hypersensitivity sites are pointed out with ball-and-sticks symbols. Conserved binding motifs are boxed. Below each autoradiograph, the corresponding nucleotide sequence is shown with annotation of the observed protection zone (red letters) and hypersensitivity sites (ball-and-stick symbols). The translational start codon is indicated with an arrow below the sequence, while the transcription start site (TSS) is pointed out with an arrow above the sequence. The TSS of *ytrA*_*Sa*_ is based on a high-resolution transcriptome database (Cohen et al., 2016), while that of *Saci_2078* is predicted based on sequence alignment of both promoter regions. Putative factor B recognition element (BRE) and TATA box promoter elements are boxed. **(B)** Sequence logo representation of a nucleotide sequence alignment of both target promoter regions, with indication of BRE, TATA box, start codon (SC) and TSS.

Subsequently, DNase I footprinting was performed to delineate the exact binding regions (Figure 4A). Binding of YtrA_Sa_ resulted in similarly sized protected regions of 34 and 35 nucleotides (nt) for the *ytrA*_*Sa*_ and *Saci_2078* targets, respectively. In addition, for both targets hypersensitivity effects were observed that indicate an increased susceptibility to DNase I digestion upon protein binding localized in one or both outer halves of the protected regions. Nucleotides that are typified by such hypersensitivity effects become more exposed to DNase I digestion through protein-induced deformations that widen the minor groove. It can thus be concluded that YtrA_Sa_ causes a DNA bending upon interacting with its binding sites. Both binding sites have a remarkably similar location with respect to transcriptional elements in the promoter: they are located downstream of the factor B recognition element (BRE) and TATA box elements, but cover the transcription initiation site, with the respective transcription start sites (TSSs) being positioned in the center of the binding regions (Figure 4A). Locational conservation of sequence-specific YtrA_Sa_ operator sites was confirmed upon aligning both promoter region sequences, with the protected regions being characterized by a high sequence identity, which is not apparent for the up- and downstream regions, with the exception of two conserved TA bps in the TATA box element (Figure 4B).

### Definition of a YtrA_Sa_ Binding Motif

**FIGURE 5:**
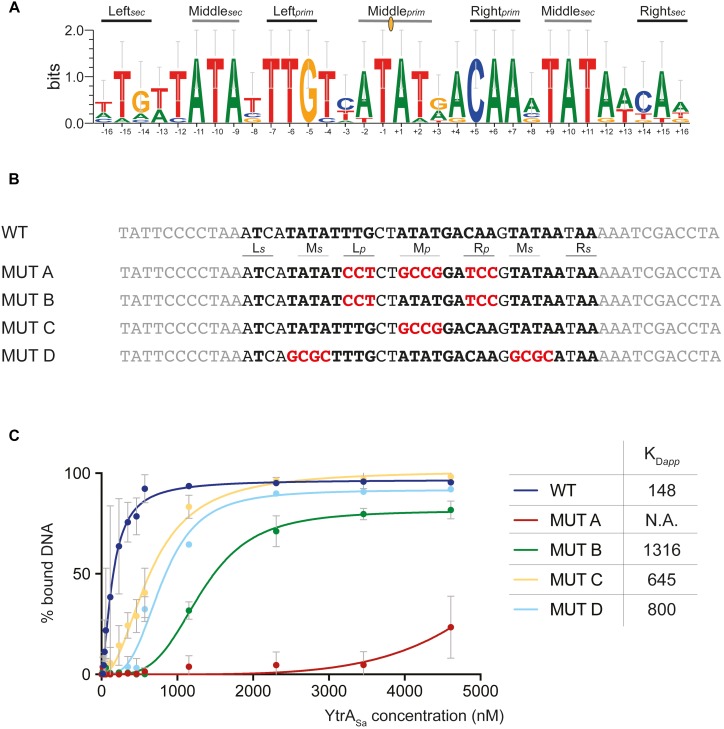
Palindromic elements in the binding motif that support YtrA_Sa_ interaction as shown by mutational analysis. **(A)** Sequence logo depicting a phylogenetic footprint using the forward and reverse sequences of the conserved YtrA binding motif identified in the *ytrA* promoter regions of *S. acidocaldarius*, *S. solfataricus*, *S. islandicus* and *A. manzaensis* and in the *Saci_2078* promoter region of *S. acidocaldarius*. Primary (prim) and secondary (sec) binding motif elements are indicated. The center of dyad symmetry is annotated with a yellow oval symbol. **(B)** Overview of the experimental design of mutated binding site variants (MUT), with the wild type (WT) binding site of the *ytrA*_*Sa*_ target as a starting point. Palindromic residues are depicted in bold and mutated residues in red. **(C)** Graphical representation of binding curve analysis based on densitometric analysis of electrophoretic mobility shift assays performed with 50-bp DNA probes containing binding site variants as presented in panel B. Averages of calculated apparent equilibrium dissociation constants (K*_Dapp_*) are shown as well for each of the variants. Experiments were performed in duplicate. K*_Dapp_* discrepancies for the 50-bp WT probe used in this experiment and the 102-bp probe used in the initial EMSA experiment shown in Figure 3 can be explained by the differing length, which affects binding affinity (Peeters et al., 2007).

Upon identifying binding sites with DNase I footprinting, it was apparent that these contained palindromic residues. To further investigate the exact nature of the recognized inverted repeat, a phylogenetic *in silico* footprinting analysis was performed using the corresponding binding sites of (putative) targets in *Sulfolobales* species that contain a YtrA_Sa_ homolog (Figure 5A). A 32-bp inverted repeat was delineated, in which different submotifs could be discerned: a 14-bp core binding motif (primary motif) with two highly conserved palindromic half sites (Left*_prim_* and Right*_prim_*) and a center that is rich in weak bp (Middle_*prim*_). On each side of the core binding motif, additional partial sites (secondary motif) were observed with a weaker conserved 3-bp semi-palindromic sequence that corresponds to the “left” and “right” half-sites of the core binding motif (Left*_sec_* and Right*_sec_*). EMSAs with 50-bp mutated variants of the *ytrA*_*Sa*_ binding motif demonstrated that each of the submotifs contributes to binding (Figures 5B,C). When mutating all primary submotifs (Left*_prim_*, Right*_prim_* and Middle*_prim_*) (MUT A), YtrA*_Sa_* binding was almost entirely abrogated, confirming the essentiality of the primary motif as a core binding site. In this core motif, the highly conserved half sites contribute more to binding affinity than the weak-bp center (compare MUT B to MUT C). Finally, as the simultaneous mutation of the secondary half sites (Middle*_sec_*) on both sides of the core binding motif (MUT D) also resulted in a strong decrease in binding affinity, a role for these secondary half sites was confirmed as well (Figure 5C).

Based on the characterized 14-bp core palindromic motif, the *S. acidocaldarius* genome was screened for additional occurrences of this binding motif, restricted to 500-bp stretches upstream of annotated ORFs (Supplementary Table S4). Several additional putative binding motif sequences were retrieved, of which two have similar *P*-values as the originally identified YtrA_Sa_ binding sites in the *Saci_2078* promoter region (between 1.0e^–06^ and 1.0e^–05^) and have an identical positioning with respect to the translational start codon by completely covering it. These binding motifs are located in the control region of *Saci_1073* encoding another membrane protein and *Saci_1554* encoding a ribosomal protein. However, analysis by EMSA and ChIP-qPCR demonstrated that neither high-affinity *in vitro* binding nor *in vivo* binding is observed for YtrA_Sa_ (Supplementary Table S4). This might be explained by a lack of conservation of secondary half sites accompanying the identified primary binding motif (Supplementary Figure S3) and suggests that these sites, given their ideal location to exert regulation and their well-conserved core motif, are either remnants of YtrA_Sa_ regulatory events that took place earlier in evolution or are targets of an alternative transcription factor with a similar DNA-binding specificity. These results confirm the restricted binding profile of YtrA_Sa_ in the *S. acidocaldarius* genome as was revealed by ChIP-seq.

### Three-Dimensional Structure of YtrA_Sa_

**FIGURE 6:**
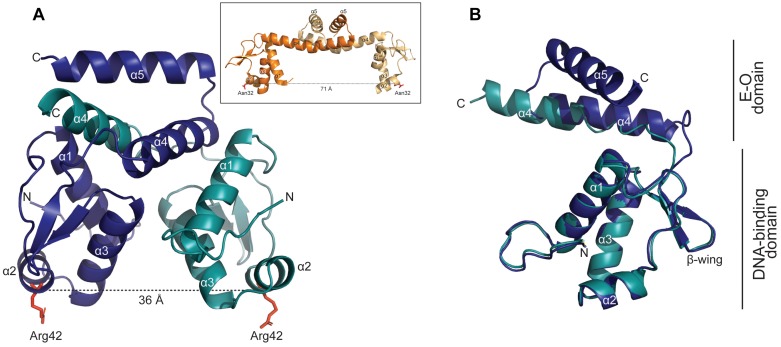
YtrA_Sa_ has a dimeric structure with a distance of 36 Å between the DNA-binding domains. **(A)** Crystallographic structure of the YtrA_Sa_ dimer with indication of the different chains in different colors (chain A: α1-5; chain B: α1-4) and the distance between two corresponding α2 residues (Arg42). The α5 helix of chain B could not be modeled due to poor electron density. In the inset, the protein structure of the YtrA-like CGL2947 of *Corynebacterium glutamicum* is displayed (PDB:2EK5) (Gao et al., 2007) with indication of the distance between conserved α2 residues. **(B)** Structural alignment of chains A and B of YtrA_Sa_ dimer with indication of the different domains.

A crystallographic analysis of YtrA_Sa_ yielded a structure at 3.0 Å resolution (Figure 6). The 14-kDa YtrA_Sa_ protein crystallized as a dimer with the asymmetric unit of the crystal structure containing a homodimer with an exclusive α-helical structure. Each monomeric unit displays an N-terminal DNA-binding domain harboring the typical GntR-like wHTH motif (α1–α3) and a short C-terminal E-O domain, consisting of two α helices (α4–α5) that mediate dimerization, which is a typical feature for YtrA-like regulators.

Upon comparing the YtrA_Sa_ structure with that of the only described bacterial YtrA-type crystal structure, CGL2947 of *C. glutamicum* (Gao et al., 2007), major structural differences are observed despite the similar topological organization (Figure 6A). The distance between the two monomeric Arg42 residues, located in wHTH domain, amounts 36 Å, which is similar to the length of one turn of the DNA helix. This is only about half of the distance between corresponding monomeric residues in the *C. glutamicum* YtrA structure (Figure 6A inset). This structural difference can be ascribed to the length of the α4 helix, which is shorter for the YtrA subfamily of which the gene is colocalized in an operon with a gene encoding a membrane protein, consisting of the *Sulfolobus* YtrA homologs and *S. coelicolor* SCO3812.

For one of the YtrA_Sa_ chains, the α5 helix could not be modeled due to poor electron density, likely explained by a flexible behavior of these C-terminal helices within the crystal. Furthermore, the alignment of individual monomeric chains from within the same dimer demonstrated that the E-O domain has an asymmetric nature (Figure 6B). Although these observations might be ascribed to the crystal packing and are not necessarily biologically relevant, the short length of α4 underlies a compact E-O domain regardless of its exact conformation and a shorter distance between the two monomeric DNA-binding domains in comparison to other YtrA-like regulators.

### Determination of the YtrA_Sa_ Regulon

**FIGURE 7:**
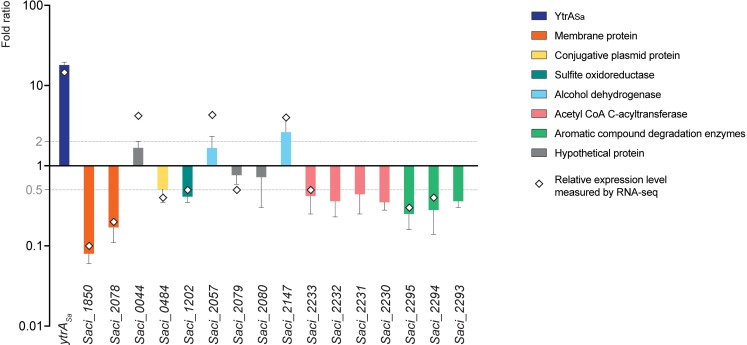
YtrA_Sa_ exerts repression on its direct target genes encoding two membrane proteins. A bar plot shows relative gene expression levels as determined by RT-qPCR, comparing a *ytrA*_*Sa*_ overexpression strain with its isogenic wild type. Error bars represent the standard deviations of three biological repeats. Average fold-change values of RNA-Seq analysis are depicted by a diamond. Gray lines represent a fold change of 2 and 0.5, respectively.

To study the effect of YtrA_Sa_ on the transcriptional expression of its target genes, a *ytrA*_*Sa*_ overexpression strain was constructed (Supplementary Figure S1) and subjected to a comparative transcriptomic analysis using an RNA-Seq approach, followed by validation with RT-qPCR (Figure 7 and Supplementary Table S5). This analysis revealed 12 differentially expressed genes, including the *ytrA*_*Sa*_ gene itself, which showed a strong upregulation in the overexpression strain, as expected. Both membrane-protein encoding genes that are targeted by YtrA_Sa_ for high-affinity binding, *Saci_1850* and *Saci_2078*, were found to be significantly downregulated in the *ytrA*_*Sa*_ overexpression strain suggesting a role as a transcriptional repressor for YtrA_Sa_.

All other genes that displayed differential expression in the *ytrA*_*Sa*_ overexpression strain, either a higher or lower expression level (Figure 7), were shown not to harbor a YtrA_Sa_ binding motif in their promoter region (Supplementary Table S4). In addition, their promoter regions did not show an interaction with YtrA_Sa_ *in vivo* or *in vitro* (Table 1, data not shown). Their differential expression can thus be explained by indirect regulatory effects. These genes belong to a variety of functional categories, such as a conjugative plasmid protein (*Saci_0484*), a sulfite oxidoreductase (*Saci_1202*), an acetyl-CoA C-acyltransferase (*Saci_2233*), encoded in an operon with other acyltransferase-encoding genes, and a predicted 4-hydroxyphenylacetate 3-hydroxylase (*Saci_2294*) and catechol 2,3-dioxygenase (*Saci_2295*), co-encoded in an operon and involved in the oxidative degradation of aromatic compounds. Analysis by RT-qPCR confirmed a slightly lower expression level for the *Saci_2230-Saci_2233* and *Saci2293-Saci_2295* operons and the sulfite oxidoreductase-encoding *Saci_0484*, but not for the other genes (Figure 7). It can thus be concluded that YtrA_Sa_ is a repressor of a restricted regulon, consisting of only two genes encoding putative membrane proteins, of which the function is unknown. Sequence analysis did not reveal any homologies to characterized membrane proteins or transporters. Possibly, these targets are transporter proteins of which the physiological function is linked to the metabolic functions of enzymes encoded by the indirectly regulated operons, such as degradation of aromatic compounds. To test this hypothesis, the effect of six aromatic compounds (phenol, catechol, biphenyl, phenylacetyl-CoA, salicylate and benzaldehyde) on the *in vitro* binding of YtrA_Sa_ to the promotor region of its own operon was analyzed by EMSA (Supplementary Figure S4). None of the compounds affected the YtrA_Sa_-DNA binding and a YrtA_Sa_ ligand is thus not identified.

## Discussion

**FIGURE 8:**
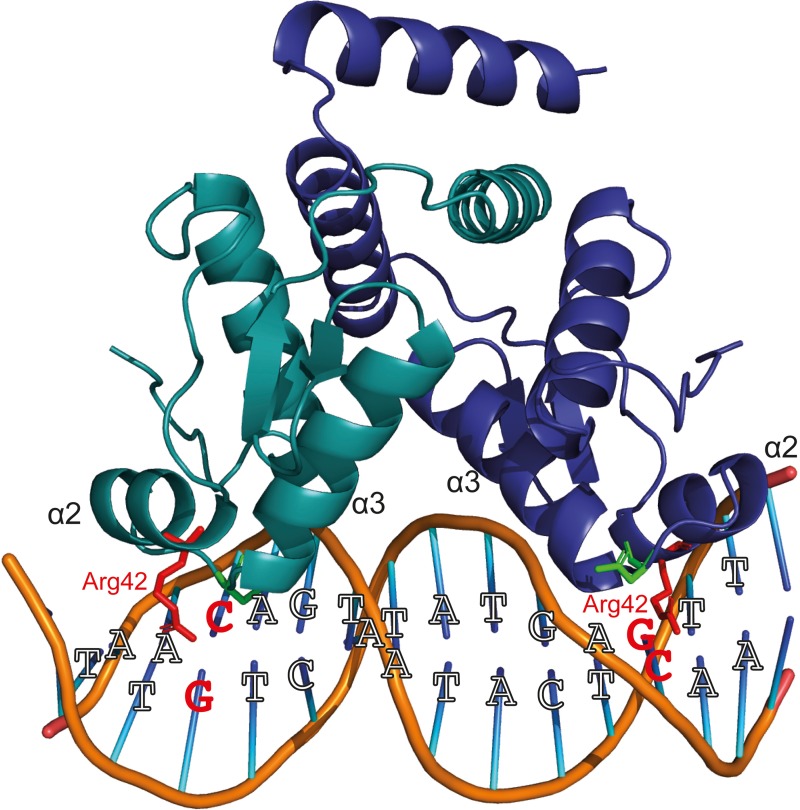
Hypothetical model of protein-DNA interactions that are possibly established when a YtrA_Sa_ dimer binds the primary consensus binding motif. This model has been built using Pymol (DeLano, 2002), with indication of the consensus bp and the wHTH helices α2 and α3. Two conserved residues presumed to be important for DNA binding (Suvorova et al., 2015), Arg42 and Asn51, are depicted in a stick representation. The Arg42 residues and GC/CG bp that are hypothesized to interact and to be essential for sequence-specific interaction are colored red.

YtrA_Sa_ performs an (auto-)repression function by interacting with high specificity and affinity with a 32-bp long inverted repeat. However, the following indications suggest that the formed YtrA_Sa_-DNA complex minimally consists of a single protein dimer that mainly establishes contacts with a core region of 14 bp, the primary binding motif: (i) protein binding assays with mutant variants of the binding site demonstrate that the primary motif is essential (Figure 5); (ii) the crystallographic dimeric structure of YtrA_Sa_ is in line with the recognition of a 14-bp binding motif, given the compact structure and corresponding residues in the two wHTH motifs being separated by a distance that roughly amounts to a single turn of the DNA helix (36 Å) (Figure 6A). A hypothetical model of the YtrA_Sa_-DNA complex shows that in such a conformation, the dimer interacts with two major groove segments and the intervening minor groove segment (Figure 8).

Sequence-specific amino acid-nucleotide interactions are presumed to take place in the major grooves that contain palindromic half sites with highly conserved bp residues, while the center of the binding motif faces with the minor groove toward the regulator and contains weak bp that facilitate protein-induced bending. In this model, the highly conserved Arg42 is ideally positioned for base-specific interactions with the symmetrical GC and CG bp in the palindromic half sites (Figure 8). This interaction is likely to occur since for bacterial YtrA-like regulators a correlation has been observed between the corresponding Arg residue and strong bp (Suvorova et al., 2015). Furthermore, an Arg interaction with GC/CG bp is known to account for a large fraction of specific protein-DNA contacts (Marabotti et al., 2008) and also occurs in FadR and HutC subfamilies of GntR TFs (Suvorova et al., 2015). This empowers our hypothesis that the half-sites in the central 14-bp motif are contacted in the major groove with the establishment of sequence-specific interactions. Interestingly, in contrast to canonical wHTH-containing DNA-binding proteins (Aravind et al., 2005), it is not the recognition helix α3 that is presumed to dock into the major groove and establish contacts with the DNA, but instead the preceding α2, which also contains the conserved Arg42 hypothesized to be involved in base-specific contacts. Indeed, the YtrA_Sa_ structure demonstrates a relative orientation of the α3 helices that precludes a potential involvement in direct DNA interactions (Figure 6).

A binding mode with a dimer interacting with a 14-bp binding motif with two-fold symmetry is often observed for prokaryotic TFs, including non-YtrA GntR members (Rigali et al., 2002; Aravind et al., 2005) and contrasts with the typically longer palindromic binding motifs of bacterial YtrA TFs (Rigali et al., 2002), such as the motif GGTG-N_16_-CACC that is recognized by a YtrA-like regulator from *Xanthomonas citri* (Zhou et al., 2017). Such a longer inverted repeat is in agreement with the protein structure described for a *C. glutamicum* YtrA TF, where the distance between the two recognition helices is 62 Å, corresponding to about two turns in the DNA helical structure (Gao et al., 2007) (Figure 6A). Aligning YtrA_Sa_ with bacterial YtrA-like TFs shows that a shorter α4 helix in the secondary protein structure leads to the more compact protein structure for the YtrA regulators in Crenarchaeota. This shorter α4 helix is also observed for a subgroup of YtrA-like TFs described in *Actinomycetes*, typically located in an operon encoding a membrane protein instead of an ABC transport system (Tsypik et al., 2016). Although no protein structure has been characterized for this subgroup of bacterial YtrA-like regulators, we can assume that they are structurally similar to the archaeal YtrA_Sa_ shown in this work. Furthermore, given that they are both (presumed to be) involved in the regulation of a membrane protein, also their functions might be related.

However, what is then the function of the secondary binding sites, located up- and downstream of the core binding motif? These contain direct repeats of each adjacent half site and have been shown to be important for YtrA_Sa_ binding as well: they are protected in footprinting experiments (Figure 4A) and have been shown to mediate the high-affinity formation of stable protein-DNA interactions *in vitro* and *in vivo* (Figure 5 and Supplementary Figure S3). Possibly, they are involved in recruiting additional YtrA_Sa_ molecules, causing an alternative oligomerization of the protein and/or mediating binding cooperativity (Figure 3). Interestingly, the existence of adjacent weaker half sites accompanying the main binding motif is commonly observed for bacterial GntR-like TFs (Suvorova et al., 2015). Although we hypothesize that the core complex consists of a dimer bound to the 14-bp primary binding motif (Figure 8), the exact stoichiometry of the YtrA_Sa_-DNA complex remains to be determined.

YtrA_Sa_ represses the transcription of an operon encoding its own gene and a membrane protein and of a second gene encoding a membrane protein by interacting with a binding site that fully covers the transcription initiation site. This binding site positioning can be presumed to lead to a direct steric hindrance of the recruitment of RNA polymerase, a repression mechanism that has been commonly observed for archaeal repressors (Peeters et al., 2013). YtrA_Sa_ has a very restricted regulon, limited to these membrane proteins, for which we were unable to predict a function. Given the analogy between YtrA_Sa_ and the *Actinomycetes* YtrA subfamily harboring a similarly short α4 helix and being co-encoded with transporter proteins (Tsypik et al., 2016), a transporter function could be hypothesized as well for the target genes of the archaeal YtrA regulators. Based on functions of differentially expressed genes when comparing the *ytrA*_Sa_ overexpression strain with its isogenic wild type, a subset of aromatic compounds as possible ligands was tested *in vitro* but none influenced the YtrA_Sa_-DNA interaction (Supplementary Figure S4). It is possible that the small E-O domain of YtrA_*Sa*_ is unable to accommodate ligand binding and only functions for dimerization of the protein as has been predicted previously for YtrA-like regulators (Yoshida et al., 2000; Rigali et al., 2002). Nevertheless, regulation might still be fine-tuned by ligand interaction by an as yet unknown ligand or by post-translational modification. The elucidation of the exact physiological function of YtrA_Sa_ will require a functional characterization of the regulated membrane proteins.

## Data Availability

The datasets generated for this study can be found in the Gene Expression Omnibus (GSE129464). The crystallographic dataset has been submitted to the Protein Data Bank (PDB: 6SBS).

## Author Contributions

LL performed the molecular cloning, protein purification, electrophoretic mobility shift assays, footprinting assays, RNA extractions, chromatin immunoprecipitation assays, qRT-PCR analysis, and data analysis. LT and FN performed the RNA-Seq and ChIP-seq analyses. ED performed the protein purification, electrophoretic mobility shift assays, and footprinting assays. KV and A-CL performed the protein purification and protein crystallography. DM performed the data analysis. EP conceived and designed the study. LL and EP wrote the manuscript.

## Conflict of Interest Statement

The authors declare that the research was conducted in the absence of any commercial or financial relationships that could be construed as a potential conflict of interest.
