# Supplementary material for: YtrASa, a GntR-Family Transcription Factor, Represses Two Genetic Loci Encoding Membrane Proteins in Sulfolobus acidocaldarius
Source: Front Microbiol. 2019 Sep 10;10:2084. doi: 10.3389/fmicb.2019.02084 (PMC6746942; doi:10.3389/fmicb.2019.02084)
Supplement: Supplementary file 1 [file Data_Sheet_1.PDF]

YtrA_{Sa}, a GntR-family transcription factor, represses two genetic loci encoding membrane proteins in *Sulfolobus acidocaldarius*

Supplementary Material

Lemmens et al.

Supplementary Table S1. Overview of all strains used in this work.

Name	Description/purpose	Reference or source
Escherichia coli DH5α	Plasmid propagation strain	Gibco
Escherichia coli Rosetta (DE3)	Protein expression host strain	Novagen
Escherichia coli B834 (DE3/pLysS)	SeMet expression host strain	Novagen
Sulfolobus acidocaldarius SK-1	Uracil auxotrophic, *ΔsuaI* strain for genetic experiments	Suzuki *et al.* 2016
Sulfolobus acidocaldarius SK-1*x*pLL1601*x*$ytrA_{Sa}$	Homologous $ytrA_{Sa}$ overexpression strain	This work
Sulfolobus acidocaldarius SK-1*x*pLL1601	Isogenic wild type of homologous $ytrA_{Sa}$ overexpression strain	This work
Sulfolobus acidocaldarius MW001	Uracil auxotrophic strain	Wagner *et al.* 2012

Supplementary Table S2. Overview of all oligonucleotides used in this work.

Name	Sequence (5' -> 3')	Purpose
EP479	CCGCGGCAACGAAACTTC	Construction of pJL1602
EP480	AATACCCTCTTATGCCTATTATATTTTTATTT AATGGTAC	Construction of pJL1602
EP481	AAAATTTGAAACTGTATACAAAGCTTTATAAA GTATTTTC	Construction of pJL1602
EP482	GCTTATTCCACTAGCAGATGTAGTAATGAGGT GAAGCTCATATG	Construction of pJL1602
EP483	CGGCCGCGTCGACCCTGCAGGGGCCATTTAGG CCAG	Construction of pJL1602
EP484	CGCTGCTAGCAATTGAGGGAGAAGGCATCATG GAGTTCCCA	Construction of pJL1602
EP485	GAATTAGGCTTAATTAAAAACGTACTTATTTA AAAGAGG	Construction of pJL1602
EP486	GATTTATAAATGCAGAATCAAAAAGTTTTTAA GGTCACTCCCATGG	Construction of pJL1602
EP487	CCATGGGAGTCACCTTAAAAAC	Construction of pJL1602
EP488	TTTTTGATTCTGCATTTATAAATCCCTCTTTT AAATAAGTAC	Construction of pJL1602
EP489	GTTTTTAATTAAGCCTAATTCTGGGAACTCCA TGATG	Construction of pJL1602
EP490	CCTTCTCCCTCAATTGCTAGCAGCGCTGGCCT AAATGGC	Construction of pJL1602
EP491	CCCTGCAGGGTCGACGCGGCCGCATATGAGCT TCAC	Construction of pJL1602
EP492	CTCATTACTACATCTGCTAGTGGAATAAGCGA AAATACTTTATAAAG	Construction of pJL1602
EP493	CTTTCTATACAGTTTCAAATTTTGTACCATTA AATAA	Construction of pJL1602
EP494	AAATATAATAGGCATAAGAGGGTATTGAAGTT TCGTTGCCGCGG	Construction of pJL1602
EP495	GCAGCGAGTCAGTGAGCGAGGAAGCCCACCGC GGCAACGAAACTTCAATAC	Construction of pJL1602
EP496	CTGGGACCAACCAAACCTAAAGCTATTTGGAA ATGAGTCCATGGGAGTCACCTTAAAAACTTTT TGATTCTG	Construction of pJL1602
EP557	CCCCCATGGGTCGACCCTGCAG	Construction of pLL1601
EP558	CCCCCATGGGAGTCACCTTAAAAACTTTTTG	Construction of pLL1601
LL021	TGAGTTGAAGCCAGGCGATA	qRT-PCR *Saci_1851*
LL022	CCCGAAGCATGTTTCTCCAA	qRT-PCR *Saci_1851*
LL106	GGCATATGATGACAAGTATAATAAAA	Cloning of ytr_{Sa} in pET24a
LL107	AACTCGAGCTTGCTATTCAC	Cloning of ytr_{Sa} in pET24a
LL140	GATGGGAGTCTTGGACTCTAGG	EMSA/ChIP-qPCR *Saci_1851* target
LL154	CTTACCTTTTATAGTAGGTAAATAAGG	EMSA/ChIP-qPCR *Saci_1851* target
LL155	AAGCGGGGGAGAGATACCTA	qRT-PCR *Saci_1850*

LL156	CTCCACTGAACCTCCTGGAC	qRT-PCR *Saci_1850*
LL201	CATCCTGCAGGCTGACAAGTATAATAAAAATC GAC	Cloning of *ytrA*$_{Sa}$ in pLL1601
LL202	CATGCTAGCCTCAGTGGTGGTGGTG	Cloning of *ytrA*$_{Sa}$ in pLL1601
LL203	TATTCCCCTAAATCATATATTTGCTATATGAC AAGTATAATAAAAATCGACCTA	Site-directed mutagenesis *Saci_1851* binding site
LL204	TAGGTCGATTTTTATTATACTTGTCATATAGC AAATATATGATTTAGGGGAATA	Site-directed mutagenesis *Saci_1851* binding site
LL205	TATTCCCCTAAATCATATATCCTCTGCCGGAT CCGTATAATAAAAATCGACCTA	Site-directed mutagenesis *Saci_1851* binding site
LL206	TAGGTCGATTTTTATTATACGGATCCGGCAGA GGATATATGATTTAGGGGAATA	Site-directed mutagenesis *Saci_1851* binding site
LL207	TATTCCCCTAAATCATATATCCTCTATATGAT CCGTATAATAAAAATCGACCTA	Site-directed mutagenesis *Saci_1851* binding site
LL208	TAGGTCGATTTTTATTATACGGATCATATAGA GGATATATGATTTAGGGGAATA	Site-directed mutagenesis *Saci_1851* binding site
LL209	TATTCCCCTAAATCATATATTTGCTGCCGGAC AAGTATAATAAAAATCGACCTA	Site-directed mutagenesis *Saci_1851* binding site
LL210	TAGGTCGATTTTTATTATACTTGTCCGGCAGC AAATATATGATTTAGGGGAATA	Site-directed mutagenesis *Saci_1851* binding site
LL211	TATTCCCCTAAATCATATATCCTAGGCATGAC AAGTATAATAAAAATCGACCTA	Site-directed mutagenesis *Saci_1851* binding site
LL212	TAGGTCGATTTTTATTATACTTGTCATGCCTA GGATATATGATTTAGGGGAATA	Site-directed mutagenesis *Saci_1851* binding site
LL213	TATTCCCCTAAATCATATATTTGCTATCGTCT CCGTATAATAAAAATCGACCTA	Site-directed mutagenesis *Saci_1851* binding site
LL214	TAGGTCGATTTTTATTATACGGAGACGATAGC AAATATATGATTTAGGGGAATA	Site-directed mutagenesis *Saci_1851* binding site
LL244	TCGTCAACAACCTATATAAGCCATA	EMSA/ChIP-qPCR *Saci_2078* target
LL245	CTATGGAGATTAGCGGGTAGAG	EMSA/ChIP-qPCR *Saci_2078* target
LL246	AACTGCGGTGAATAAAGTAGGG	EMSA/ChIP-qPCR *Saci_1554* target
LL247	TGGTCACAATGTATGCTACCAA	EMSA/ChIP-qPCR *Saci_1554* target
LL248	GGGAAAGGATATTGGGGAAA	EMSA/ChIP-qPCR *Saci_0421* target
LL249	GAGATTATTGTTGCAAGGATTACA	EMSA/ChIP-qPCR *Saci_0421* target
LL250	GTTTTGAGTACAACCTAACCCATT	EMSA/ChIP-qPCR *Saci_2168* target
LL251	TTGCAGACCTTTTCCCTCAA	EMSA/ChIP-qPCR *Saci_2168* target
LL252	TCCTGAAGGCCTAAAAGAGTACA	EMSA/ChIP-qPCR *Saci_0677* target
LL253	AATCACTTCTTTAATGAAAAGTTTGAG	EMSA/ChIP-qPCR *Saci_0677* target
LL254	AGCAACACCAAAGGATTGAA	EMSA/ChIP-qPCR *Saci_0871* target
LL255	TCTTCCTACGTTCACGGTTTG	EMSA/ChIP-qPCR *Saci_0871* target
LL256	AAAATGGGAGAGTAATCGGTTTT	EMSA/ChIP-qPCR *Saci_1234* target
LL257	ACGCCTCGCTTTATTTCCAT	EMSA/ChIP-qPCR *Saci_1234* target
LL288	CCTTCCGAACTACCTGTTGC	qRT-PCR *Saci_2078*
LL289	GATAGGGCGGAAGAAAAACC	qRT-PCR *Saci_2078*
LL290	TATTCCCCTAAATCAGCGCTTTGCTATATGAC AAGGCGCATAAAAATCGACCTA	Site-directed mutagenesis *Saci_1851* binding site
LL291	TAGGTCGATTTTTATGCGCCTTGTCATATAGC AAAGC GCTGATTTAGGGGAATA	Site-directed mutagenesis *Saci_1851* binding site
LL292	TATTCCCCTAAATCAGCGCTTTGCTATATGAC AAGTA TAATAAAAATCGACCTA	Site-directed mutagenesis *Saci_1851* binding site
LL293	TAGGTCGATTTTTATTATACTTGTCATATAGC AAAGC	Site-directed mutagenesis *Saci_1851* binding site

	GCTGATTTAGGGGAATA	
LL294	TATTCCCCTAAATCATATATTTGCTATATGAC AAGGC GCATAAAAATCGACCTA	Site-directed mutagenesis *Saci_1851* binding site
LL295	TAGGTCGATTTTTATGCGCCTTGTCATATAGC AAATATATGATTTAGGGGAATA	Site-directed mutagenesis *Saci_1851* binding site
LL366	CCAGGAGCGAAGTGGGTTAT	qRT-PCR *Saci_1202*
LL367	GGATAAACGGTCTAGCGGGA	qRT-PCR *Saci_1202*
LL368	GTTACAGGAGTGCTATTCTAAG	EMSA *Saci_1202* target
LL369	CACATACTTTTGTCCTGGTG	EMSA *Saci_1202* target
LL370	GGATACGAGGAAGGACAGCA	qRT-PCR *Saci_2295*
LL371	CGTCTTAAGCCCACTAGCCT	qRT-PCR *Saci_2295*
LL372	GATGAACAGCTTTCATACTTTTTAG	EMSA *Saci_2295* target
LL373	CTGACACAAACATGCGATAG	EMSA *Saci_2295* target
LL374	GTACTGCGGGACCTTATGCT	qRT-PCR *Saci_2294*
LL375	TCTCCCTCGGATAGAAGCCT	qRT-PCR *Saci_2294*
LL376	GTCTATGCCCCTAAGAGTGGT	qRT-PCR *Saci_2293*
LL377	GATCCCTCCTCCTCGCATAA	qRT-PCR *Saci_2293*
LL378	CAAGACGACCGGGCAATTAG	qRT-PCR *Saci_0484*
LL379	CGACGAACAACTGCCACATA	qRT-PCR *Saci_0484*
LL380	GATGACAGTGTAGGGACAAAC	EMSA *Saci_0484* target
LL381	GTCCGGAAACTCAATTATTTGTTC	EMSA *Saci_0484* target
LL382	TGACCCGCACTATCTGACTT	qRT-PCR *Saci_0044*
LL383	ACCCGAACCACATCCACAAT	qRT-PCR *Saci_0044*
LL384	GTATTGATGATTTATAATAATATAGTCG	EMSA *Saci_0044* target
LL385	CAGTTTTATTTAAAATAAAATTGAACTG	EMSA *Saci_0044* target
LL386	GAGAAGGGATTGGGAGGGTT	qRT-PCR *Saci_2147*
LL387	TAGGGCTTGCATGGTGGTTA	qRT-PCR *Saci_2147*
LL388	CACGTAAAATTTATCATTGTATTATTAAC	EMSA *Saci_2147* target
LL389	GTATTGCCGCCTTGTATAC	EMSA *Saci_2147* target
LL390	AGCCTCACCTTTAGCCTGTT	qRT-PCR *Saci_2057*
LL391	CTTCCGTGAATAGCCTTGGC	qRT-PCR *Saci_2057*
LL392	GTGTAGTTATATGAAACATGTTCTAC	EMSA *Saci_2057* target
LL393	GTAAGGGCTCACCGAATTTATG	EMSA *Saci_2057* target
LL394	GTATTTGAGGCAGCGTCGAG	qRT-PCR *Saci_2233*
LL395	AACCCTGGATTGTATCGCCT	qRT-PCR *Saci_2233*
LL396	CATATTATTTCCAAATATGTTGAAAAATAC	EMSA *Saci_2233* target
LL397	CAATAATGGCTGTTCTATTC	EMSA *Saci_2233* target
LL398	TGGAGGAGCAGCAATGGTTA	qRT-PCR *Saci_2232*
LL399	CTCTAGCGGGCCATTCTGTA	qRT-PCR *Saci_2232*
LL400	TGCGGCAAGATTTACGTTCC	qRT-PCR *Saci_2231*
LL401	TGCCGTCATTACATAGCCCT	qRT-PCR *Saci_2231*
LL402	GGGAAAGTCTATCTACCGCCA	qRT-PCR *Saci_2230*
LL403	ACAGACCGCCATTTACACCT	qRT-PCR *Saci_2230*
LL404	GTGGGTAACTGGGCTACTGT	qRT-PCR *Saci_2079*
LL405	TCTTCAACTCCTCTGCAGCT	qRT-PCR *Saci_2079*
LL406	CATAGTTGTCCAGAGGTTG	EMSA *Saci_2079* target
LL407	CTTCCCTCCTAACCTTAGTG	EMSA *Saci_2079* target
LL408	AACTGGGGAACGTTTTGTGG	qRT-PCR *Saci_2080*
LL409	AGTTACCAGGTAAGCAGCGT	qRT-PCR *Saci_2080*
LL410	TTGAAACTATCGGAAAGAGCA	ChIP-qPCR *Saci_0894* target
LL411	TCCGTCAGCATCAATAACCA	ChIP-qPCR *Saci_0894* target
LL412	TTTGAATGAGCTGTCGAACG	ChIP-qPCR *Saci_0505* target

LL413	TCCAGTACAGGAAGGGCAAC	ChIP-qPCR *Saci_0505* target
LL414	AAGGTTTTTATGAACCCTTTTCTTG	ChIP-qPCR *Saci_1323* target
LL415	TTATATGAAGGTTGATTTGAAGACA	ChIP-qPCR *Saci_1323* target
LL416	ACAGCAAGTGGTTCATCACG	ChIP-qPCR *Saci_2319* target
LL417	TGTTCTACCGGCAGGGATAG	ChIP-qPCR *Saci_2319* target

Supplementary Table S3. Overview of all plasmids used in this work.

**Name**	**Description/purpose**	**Reference or source**
pET24a	Expression vector	Novagen
pET24a*xytrA*$_{Sa}$	YtrA$_{Sa}$ overexpression vector	This work
pSVA1450	*S. acidocaldarius/E.coli* shuttle vector with P_{mal} upstream of *lacS* reporter	Wagner *et al.* 2012
pJL1602	pSVA1450 variant with constitutive *lacS* expression	This work
pLL1601	pSVA1450 variant with constitutive *lacS* expression and P_{mal} upstream of MCS	This work
pLL1601*xytrA*$_{Sa}$	Homologous *ytr*$_{Sa}$ overexpression strain	This work

Supplementary Table S4. Analysis of additional *in silico* predicted targets (based on a genome screening with the $YtrA_{Sa}$ primary binding motif).

Gene number	Annotation	Predicted binding motif	*p*-value	Position to startcodon (start/end)		*In vitro* binding?	*In vivo* binding?
Saci_1851	$YtrA_{Sa}$	TTGCTATATGACAA	7.4E-06	-7	+7	Yes	Yes
Saci_2078	Membrane protein	TTGTTATATGACAA	3.0E-05	-7	+7	Yes	Yes
Saci_1073	Membrane protein	TTGCCTTATGGCTA	9.3E-05	-7	+7	Low affinity	No
Saci_1554	30S ribosomal protein	TTGATTTTTGCCAA	2.1E-05	-7	+7	Low affinity	No
Saci_0421	Membrane-bound transferase	TTGTGTTTAAACAA	4.0E-05	-2	+12	No	No
Saci_2168	Glutamyl-tRNA aminotransferase subunit	TTGGGAAAGTGCAA	1.0E-04	-8	+6	No	No
Saci_0677	Site-specific recombinase XerD	TAGGCATAATGCAA	1.0E-04	-40	-26	No	No
Saci_0871	Zn-dependent protease	TTGACTTAAAGCAA	2.1E-05	-61	-47	No	No
Saci_1234	Mannose-6-phosphate isomerase	TTGAGACTTCGCAA	6.2E-05	-24	-10	No	No

Supplementary Table S5. Summary of RNA-seq data (*ytrA$_{Sa}$* overexpression strain versus isogenic wild type (WT)). Log FC = log of the fold change of expression in *ytrA$_{Sa}$* overexpression strain versus WT; F = the empirical Bayes quasi-likelihood F-test statistic; *p*-value: associated *p*-value; FDR = false discovery rate. N.A. = not applicable.

Gene number	Annotation	Log FC	F	*p*-value	FDR
Saci_1851	YtrA$_{Sa}$	-3.87	900.23	2.06E-09	4.58E-06
Saci_1850	Predicted membrane protein	2.91	738.72	4.47E-09	4.96E-06
Saci_1202	Sulfite oxidase-like oxidoreductase	1.03	134.00	3.17E-06	2.15E-3
Saci_2294	4-hydroxyphenylacetate 3-hydroxylase	1.30	116.42	5.36E-06	2.15E-3
Saci_0484	Conjugative plasmid protein	1.21	113.98	5.80E-06	2.15E-3
Saci_2078	Predicted membrane protein	2.39	93.50	1.20E-05	3.82E-3
Saci_0044	CBS domain containing protein	-2.07	80.18	2.11E-05	4.51E-3
Saci_2147	Aspartate semialdehyde dehydrogenase	-1.99	67.93	3.83E-05	6.55E-3
Saci_2295	3,4-dihydroxyphenylacetate 2,3-dioxygenase	1.60	57.48	6.93E-05	7.75E-3
Saci_2057	NAD(P)-dependent alcohol dehydrogenase	-2.10	54.62	8.28E-05	8.37E-3
Saci_2233	Acetyl-CoA acetyltransferase	1.04	48.59	1.24E-04	9.20E-3
Saci_2079	Hypothetical protein	1.06	47.15	1.14E-04	9.88E-3
Saci_RS11760	Hypothetical protein	-1.71	119.59	4.85E-06	2.15E-3
Saci_RS07300	Integrase	2.05	87.15	1.56E-05	4.33E-3

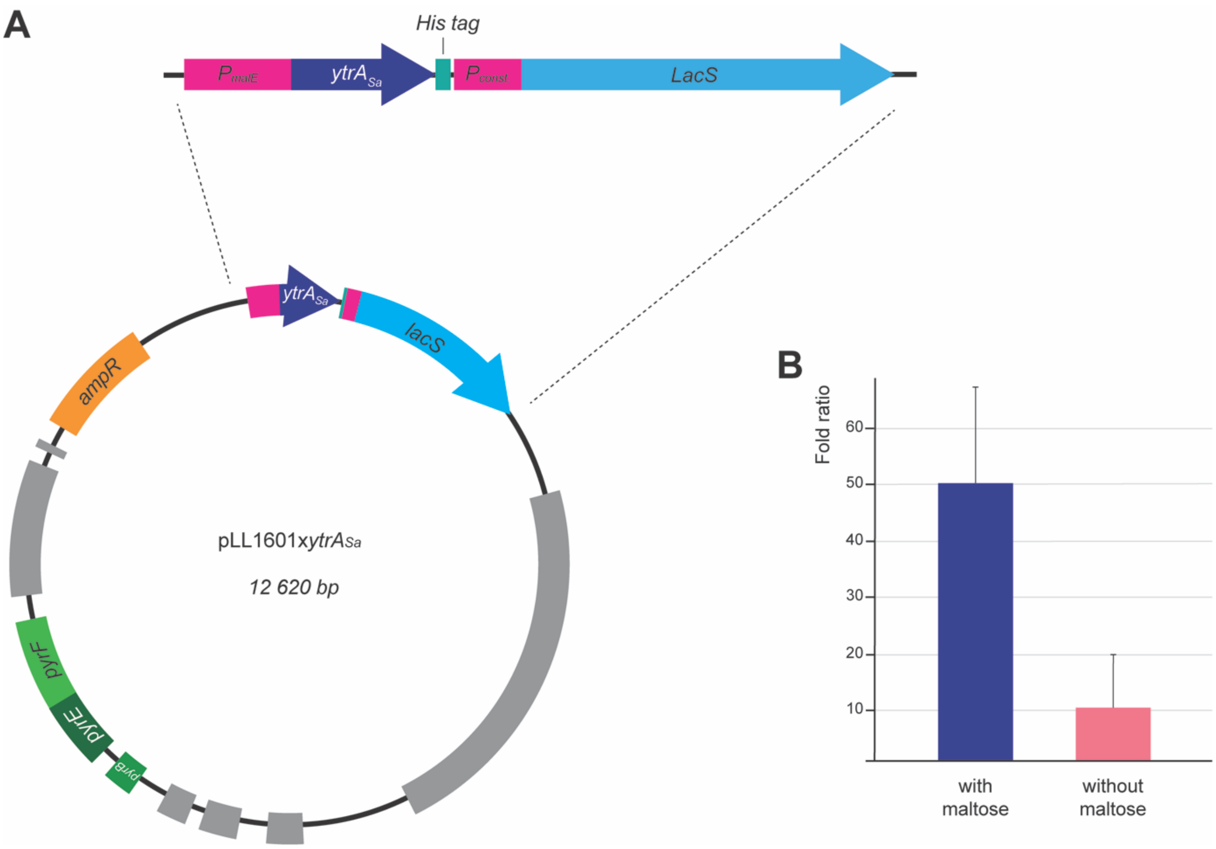

Supplementary Figure S1. Construction of a *ytrA*$_{Sa}$ overexpression strain. A. Schematic depiction of the overexpression construct pLL1601*xytrA*$_{Sa}$, which is derived from pSVA1450 as described in the Materials and Methods section. **B**. Relative gene expression analysis of *ytrA*$_{Sa}$ using RT-qPCR in a genetic background harbouring pLL1601*xytrA*$_{Sa}$ versus one that harbours an empty pLL1601 plasmid, in inducing (addition of 0.4% maltose) and non-inducing conditions.

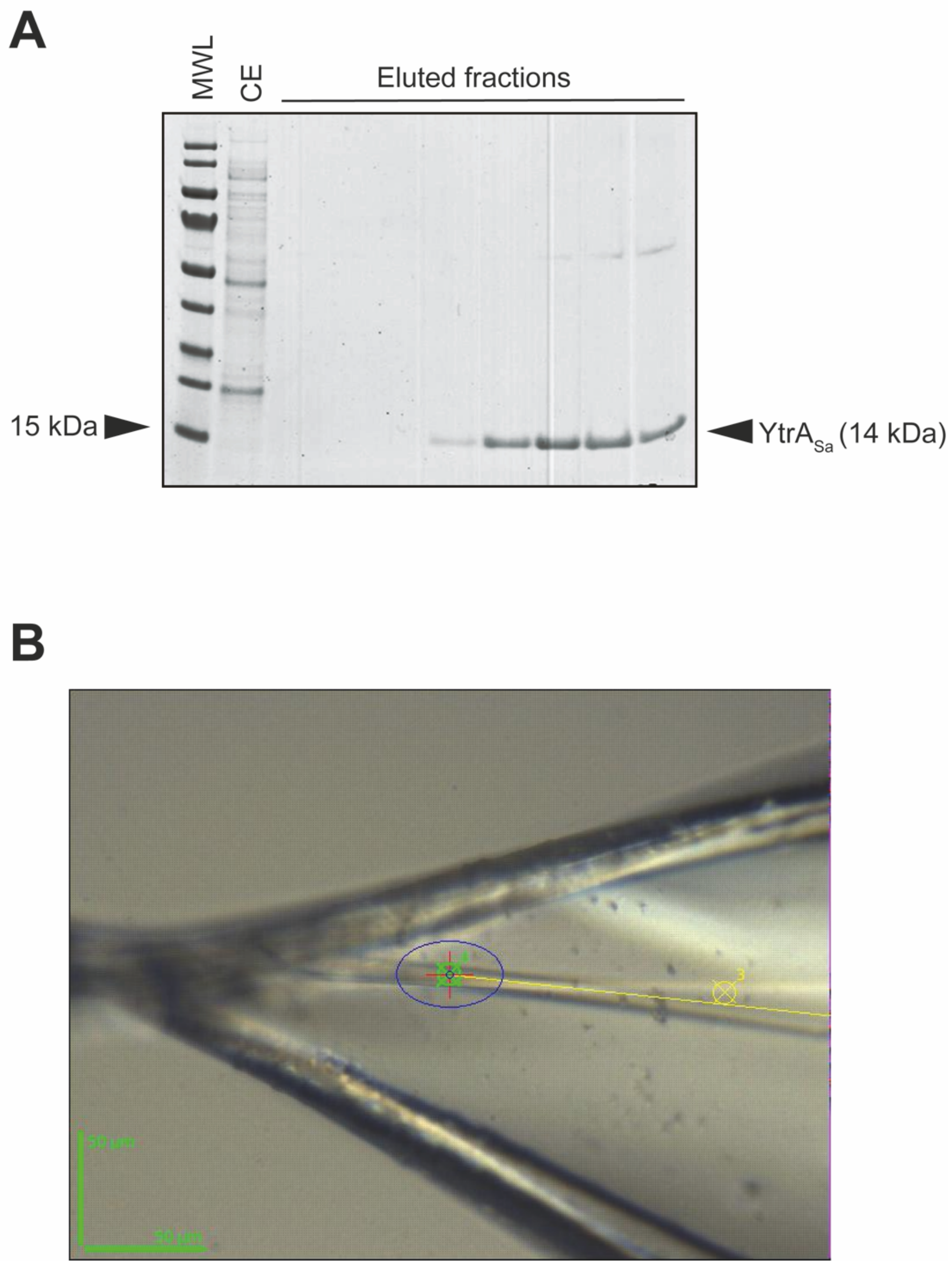

Supplementary Figure S2. Purification and crystallization of YtrA$_{Sa}$ protein. A. SDS-polyacrylamide gel electrophoresis of eluted fractions after His-tag affinity chromatography. MWL = molecular weight ladder; CE = crude extract. **B**. Picture of thin needle-shaped crystals of YtrA$_{Sa}$ protein that were used to obtain a diffraction pattern.

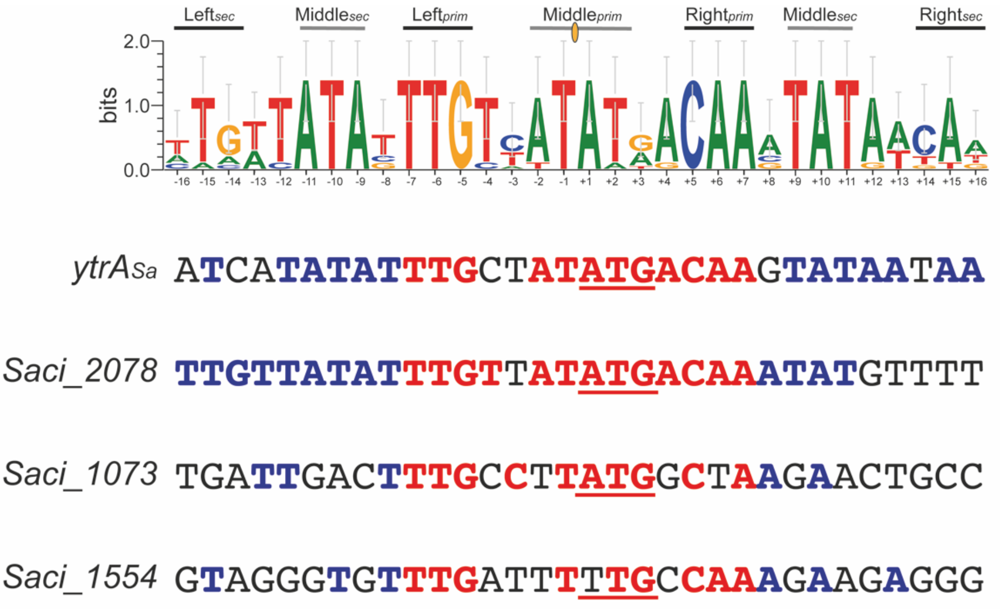

Supplementary Figure S3. Additional predicted YtrA$_{Sa}$ binding motifs. A sequence logo depicting a phylogenetic footprint of the YtrA$_{Sa}$ binding motif is shown above the two characterized YtrA$_{Sa}$ binding motif sequences (*ytrA$_{Sa}$* and *Saci_2078*) and the two predicted motifs (*Saci_1073* and *Saci_1554*) (Supplementary Table S4). Conserved residues of the core primary binding motif are depicted in red, conserved residues of the secondary half sites in blue. The translational start codon is underlined.

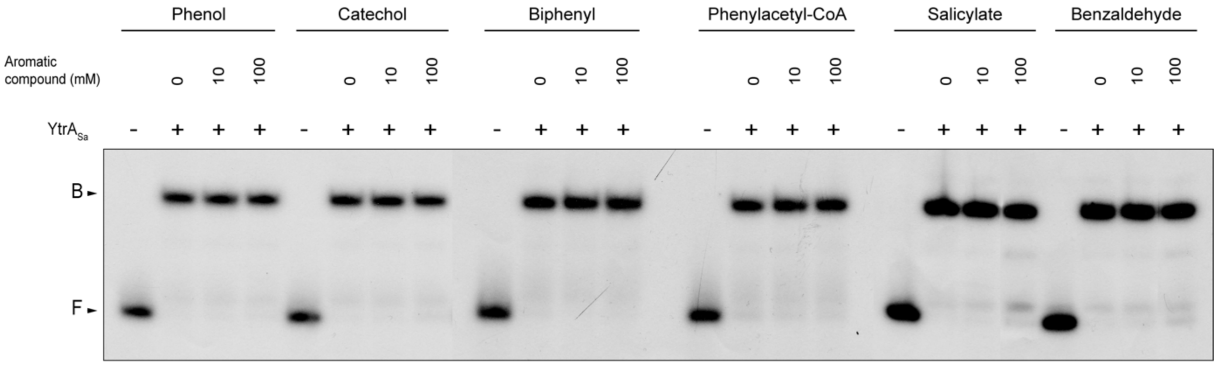

Supplementary Figure S4. Aromatic compounds do not affect YtrA$_{Sa}$-DNA interactions. Electrophoretic mobility shift assays (EMSAs) of YtrA$_{Sa}$ binding to a radiolabeled DNA probes of about 100 bp representing the control region of the *Saci_1850-Saci_1851* operon in the presence of different concentrations of aromatic compounds. "+" indicates the addition of 460 nM YtrA$_{Sa}$. Populations of free DNA (F) and YtrA$_{Sa}$-bound DNA (B) are indicated with an arrowhead.

Supplementary references

Suzuki, S. & Kurosawa, N. (2016). Disruption of the gene encoding restriction endonuclease SuaI and development of a host-vector system for the thermoacidophilic archaeon *Sulfolobus acidocaldarius*. *Extremophiles* **20**(2), 139-148.

Wagner, M., van Wolferen, M., Wagner, A., Lassak, K., Meyer, B.H., Reimann, J. & Albers S.-V. (2012). Versatile genetic tool box for the crenarchaeote *Sulfolobus acidocaldarius*. *Frontiers in Microbiology* **3**, 214.
